# Differences in Interfacial Reactivity of Graphite and Lithium Metal Battery Electrodes Investigated Via Operando Gas Analysis

**DOI:** 10.1021/acs.jpcc.4c03656

**Published:** 2024-08-06

**Authors:** J. Padmanabhan Vivek, Nuria Garcia-Araez

**Affiliations:** † Chemistry, University of Southampton, Southampton SO17 1BJ, United Kingdom; ‡ The Faraday Institution, Harwell Campus, Didcot OX11 0RA, United Kingdom

## Abstract

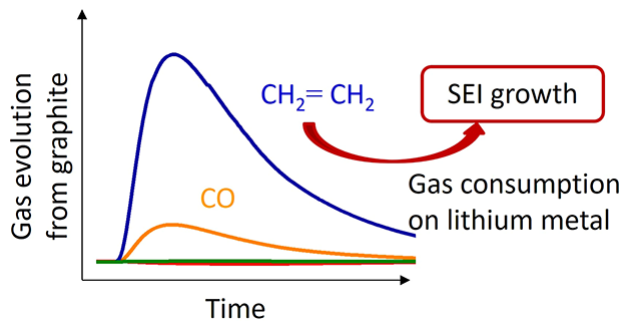

Gases evolved from lithium batteries can drastically affect their performance and safety; for example, cell swelling is a serious safety issue. Here, we combine operando pressure measurements and online electrochemical mass spectrometry measurements to identify the nature and quantity of gases formed in batteries with graphite and lithium metal electrodes. We demonstrate that ethylene, a main gas evolved in SEI formation reactions, is quickly consumed at lithium metal electrodes unless they have been pretreated in the electrolyte. Polyolefins such as polyethylene are suggested as the possible reaction product from ethylene consumption, evidencing another pathway of SEI formation that had been previously overlooked because it does not produce any gas product.

## 1 Introduction

Excessive gassing of lithium-ion batteries severely compromises performance and safety. The key importance of understanding gas formation in batteries is highlighted by the very costly recalls of faulty batteries, due to swelling and other issues, that have been undertaken by many companies.^1−3^

Furthermore, the characterization of gas evolution from batteries also contributes hugely to deepen the understanding of battery reactions to guide performance and safety improvements.^4−11^ Particularly for graphite and lithium metal anodes, which are the most important anode materials for current and next-generation batteries, the investigation of the evolution of gases provides unique insights into the reactions involved in the formation of the solid electrolyte interphase (SEI).^12−17^

Previous studies have shown that the main cause of capacity fade in lithium-ion batteries is the occurrence of slow side reactions at the graphite electrode, which irreversibly consume the lithium inventory.^18−24^ These side reactions take place because of the limited stability or protective efficiency of the graphite SEI; thus, the investigation of the graphite SEI is one of the most important areas in battery research.^25−29^ Similarly, the investigation of the formation of the SEI on lithium metal anodes is critical for the development of high energy lithium metal anode batteries as well as for improving the understanding of lithium plating reactions that severely limit the lifetime of graphite-based lithium-ion batteries.^30−33^ However, the current understanding of these complex reactions is limited, and little is known about the differences in the SEI reaction mechanism and gas formation properties of graphite and lithium metal anodes.

In this work, we combine operando pressure measurements and online electrochemical mass spectrometry to investigate the gases evolved and consumed in batteries containing graphite and lithium metal electrodes. By comparing the gas formation properties of graphite in a lithium half-cell and in a cell with a LiFePO_4_ counter electrode, we demonstrate that the lithium counter electrode in the half-cell leads to a significant consumption of gases over time. The operando analysis of gases via mass spectrometry evidences that ethylene (C_2_H_4_) is more quickly consumed at the lithium electrode than at the graphite electrode. While the formation of ethylene (C_2_H_4_) is often used as a signature of SEI (re)formation reactions,^34−40^ this work highlights that C_2_H_4_ can take part in further reactions and thus it might not be quantitatively released to the cell headspace. The present results also demonstrate the risk of misinterpreting gas analysis results obtained in half-cells when the intrinsic reactivity of lithium electrodes is not taken into account accurately.

## 2 Methods

### 2.1 Electrode Preparation and Cell Assembly

For the operando pressure measurements and online electrochemical mass spectrometry (OEMS) measurements, the electrodes were coated on a fine steel mesh (SS316 grade, the Mesh Company) to allow better gas diffusion from both sides of the electrode. Graphite electrodes were prepared by mixing the active material powder (mesophase MGP-A graphite, China Steel Chemical Corp), poly(vinylidene difluoride) (PVDF 5130, Solvay), and Super C65 conductive carbon black (Timcal), in 94:3:3 mass ratio, and *N*-methyl-2-pyrrolidone (NMP, Sigma-Aldrich, 99.5%, anhydrous) was added to this to form an ink. The ink was mixed in a planetary mixer (Thinky ARE-250) three times at 2000 rpm for 5 min, with 5 min breaks in between for cooling. The slurry was then blade-coated on a fine steel mesh using an automatic film coater (MTI, MSK-AFA-III) to a wet thickness of 180 μm, producing a graphite loading of ca. 5 mg cm^–2^. Prior to coating, the steel mesh was calendared to remove creases; an aluminum foil was placed under the mesh during doctor-blading. In a similar way, lithium iron phosphate (LiFePO_4_) counter electrodes were prepared by mixing LiFePO_4_, PVDF, and Super C65 carbon in a 91:4:5 mass ratio and the slurry was coated on a fine steel mesh to a wet thickness of 450 μm. The slurry coated mesh was then transferred to a vacuum oven and dried at 80 °C for 12 h. The electrodes were punched in discs of 25 mm using a hand-held precision punch (Nogami, Japan) and then pressed using a hydraulic pellet press (Specac) at 5 tonne pressure. The electrodes were further dried for 48 h in a Buchi glass vacuum oven (6 h at 25 °C, 8 h at 80 °C, 12 h at 100 °C, and then 22 h at 120 °C), and then, the sealed glass oven was transferred to an argon filled glovebox (MBraun, Germany; O_2_ and H_2_O < 1 ppm). In a similar way, Glass Fiber B separator and LiFePO_4_ counter electrodes (where applicable) were also cut to 25 mm discs and then dried and transferred to the glovebox. All the Swagelok cell components were dried under vacuum at 80 °C for 12 h.

The electrolyte was 1 M LiPF_6_ in a mixture of ethylene carbonate (EC) and ethyl methyl carbonate (EMC) in a 3:7 ratio by weight (LP57, Soulbrain), and the water content, determined by Karl Fischer titration, was <5 ppm.

### 2.2 Operando Pressure Measurements

Operando pressure measurements were conducted to quantify the amount of gases evolved in battery reactions, using a Swagelok cell design with low headspace volume that provides very high sensitivity.^41^ A pressure transducer (PA-33X, Keller Druck AG) was used to monitor the internal pressure of the cell. Copper or aluminum plungers were used for the lithium or LiFePO_4_ counter-electrode side, and a perforated steel plunger, connected to the pressure transducer, was used on the graphite electrode side. The cells were assembled inside an argon filled glovebox (O_2_ and H_2_O < 1 ppm) as follows: a 25 mm lithium foil disc was placed on the copper current collector at the base of the cell; then, 200 μL electrolyte was added to the center of the lithium disc, then a Glass Fiber B separator was placed on top of this, and another 200 μL electrolyte was added to the center of the separator; then, the graphite disc electrode was placed on top of this ensuring proper alignment of the electrodes and the separator. The steel current collector was then placed on top of the graphite electrode, and the sealed cell was brought outside of the glovebox, further tightened to ensure sealing, and then transferred to a climatic chamber set to 25 °C. Graphite/LiFePO_4_ cells were assembled in a similar manner, but a LiFePO_4_ electrode was used in place of the lithium electrode. In some experiments, the lithium electrode was soaked in the electrolyte for 24 h prior to cell assembly. In this case, the presoaked lithium electrode was carefully transferred to the cell, and then the cell was assembled with the procedure explained above, using fresh electrolyte. Electrochemical measurements were performed using a Biologic MPG2 potentiostat/galvanostat instrument running EC-lab software. The cells were allowed to rest at 1.5 V vs Li^+^/Li (at −2 V vs LiFePO_4_ for graphite/LiFePO_4_ cells) for 6 h, except for cells assembled using presoaked lithium electrodes, where the cells were allowed to rest for 48 h. The rest period allowed the cells to achieve a stable temperature and pressure, and then the cells were cycled between 1.50 V and 5 mV vs Li^+^/Li (between −2.0 V and −3.445 V vs LiFePO_4_ for graphite/LiFePO_4_ cells) in constant current mode.

### 2.3 Online Electrochemical Mass Spectrometry Measurements (OEMS)

OEMS experiments were conducted to identify which gases were evolved from graphite electrodes during charging. The OEMS setup consists of a quadrupole mass spectrometer (Pfeiffer Thermostar) connected to a specially designed electrochemical cell and a 50 μm capillary of the mass spectrometer was connected to the electrochemical cell via a manual GC sampling valve (Valco). A Swagelok electrochemical cell with an inlet and outlet drilled through the working electrode (in this case, graphite) current collector was used for OEMS studies. The outlet of the electrochemical cell was connected to the mass spectrometer capillary via the GC sampling valve. The inlet of the electrochemical cell is connected to a pressure controller (EL-Press, Bronkhorst) that is set to maintain the pressure inside the electrochemical cell equal to 1.15 bar (with 0.5% full-scale accuracy and a 500 ms response time). Between the inlet of the electrochemical cell and the pressure controller, a 3-way valve (Swagelok) connected to a vacuum pump allowed vacuum purging of the gas lines and thus contaminant-free transfer of the electrochemical cell to the OEMS setup. The outlet of the electrochemical cell had a quick disconnect double shut-off valve assembly (Beswick Engineering, USA), which connects to the GC sampling valve, and any dead volume of air trapped between the internal and external valve assembly was purged out by flowing argon through the outlet valve of the GC sampling adapter. The capillary connected to the mass spectrometer and the capillary inlet were heated to 120 °C to prevent solvent condensation. The flow of gases from the cell to the mass spectrometer is limited to ca. 9 μL/min by the dimensions of the capillary (50 μm diameter, 1 m length). This design of the OEMS system minimizes argon gas flow through the electrochemical cell and minimizes solvent evaporation.^42^ For quantification of the gas evolution rates, the setup was calibrated for H_2_, C_2_H_4_, CO, and CO_2_ (*m*/*z* values of 2, 26, 28, and 44, respectively) using standard calibration gases of known concentrations (SIP Analytical). Two calibration gas cylinders, one containing H_2_, C_2_H_4_, O_2_, and CO_2_ (each 1000 ppm in Ar) and the other one containing 1000 ppm of CO and H_2_ in Ar, were used separately to avoid overlap of the fragments, following previous work by Gasteiger’s group.^43^ The C_2_H_4_ mass spectrum has three main signals at *m*/*z* values of 28, 27, and 26, and the *m*/*z* = 26 signal was employed to determine its concentration so as to avoid interference from the CO signal at *m*/*z* = 28 and from the EMC solvent vapor at *m*/*z* = 27.^44^ Using the first calibration gas, the ratio of the *m*/*z* = 26 and *m*/*z* = 28 signals due to C_2_H_4_ was determined, which was then used to correct the contribution from C_2_H_4_ to the measured signal at *m*/*z* = 28, and the second calibration gas was then used to correlate the thus corrected *m*/*z* = 28 signal to the CO concentration. The EMC solvent vapor also gives a signal contribution at *m*/*z* = 28, but since the pressure inside the cell was maintained constant, with the pressure controller, such contribution also remained constant.

## 3 Results and Discussion

**Figure 1:**
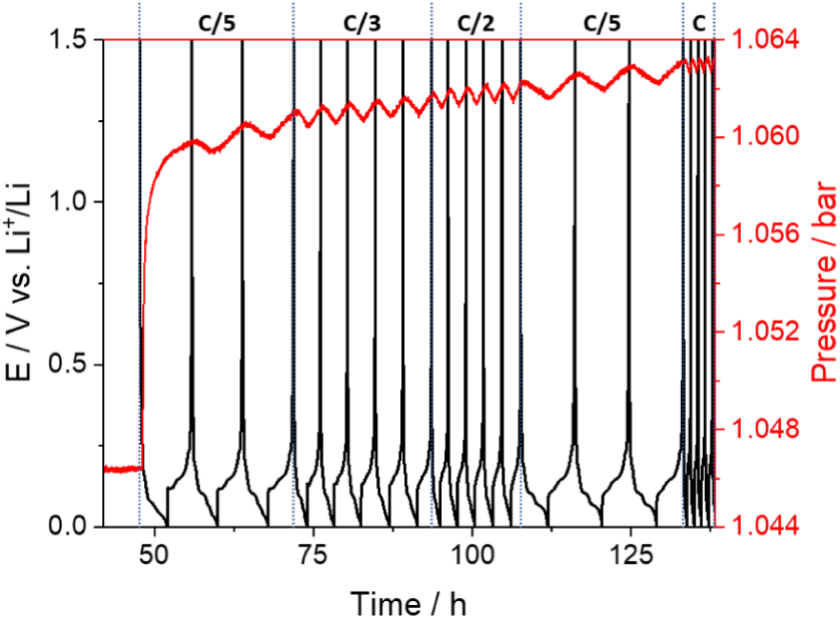
Operando pressure measurements of a graphite vs lithium cell. Prior to the measurements, the lithium electrode had been soaked in electrolyte for 24 h, and additionally, the cell was left for equilibration with the graphite at 1.5 V vs Li^+^/Li for 48 h.

The quantification of the amount of gas evolved from graphite electrodes in the SEI formation process can be achieved via operando pressure measurements, which we performed with a cell setup with low headspace volume that provides very high sensitivity in the gas detection (see the cell sketch in Figure S1).^41^Figure 1 shows the evolution of the internal pressure of the cell during cycling of a graphite electrode (mesophase MGP-A graphite, China Steel Chemical Corp) in a lithium half-cell. The sudden increase in cell pressure in the first charge cycle of graphite is due to the buildup of gases, formed in the SEI formation process, inside the cell headspace. The volume of gas generated, Δ*V*, can be calculated from
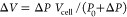
1where Δ*P* is the change in pressure in the cell (in this case, 0.016 bar), *P*_0_ is the initial pressure (in this case, 1.047 bar), and *V*_cell_ is the cell headspace volume (in this case, 2.55 mL). The calculation gives a volume of gas normalized by the mass of graphite of 1.6 mL_gas_/g_graphite_, in reasonable good agreement with the value of 2.2 mL_gas_/g_graphite_ reported by us for MAG Hitachi graphite^41^ and with the value of 2 mL_gas_/g_graphite_ reported by Gasteiger’s team for SLP30 Timcal graphite.^12,45^Figure S2 shows SEM images of the mesophase MGP-A graphite electrode used here, showing a homogeneous particle size close to 20 μm, and that, after cycling, the graphite particles are covered by a porous film produced due to electrolyte degradation (SEI formation). The voltage profiles in Figure S3 show that the first cycle at C/5 produces a reversible capacity of 354 mAh g^–1^ and an irreversible capacity of 35 mAh g^–1^, in agreement with previous studies with mesophase graphite electrodes.^46^

The operando pressure measurements in Figure 1 also show the presence of cyclic changes in pressure, which are clearly visible in the second and following cycles, and that occur synchronously with the cycling, with the insertion of lithium into graphite producing a decrease in pressure and the extraction of lithium from graphite producing an increase in pressure. In our previous work,^41^ we showed that these cyclic and reversible changes in pressure are due to the volumetric changes of the electrodes, which are largely dominated by the lithium counter-electrode, and can be estimated from
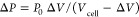
2

Under the present experimental conditions, the insertion of lithium into graphite is estimated to produce a change in electrode volume of 1.3 μL, based on the expansion of the crystallographic structure of 13.2% obtained from XRD measurements,^47^ and the coupled electrochemical reaction of oxidation of the lithium counter electrode is estimated to produce a change in electrode volume of −4.4 μL (see details of calculations in the Supporting Information). These effects combined produce an expected change in pressure, calculated with eq 2, of −1.3 mbar, in good agreement with the experiments.

**Figure 2:**
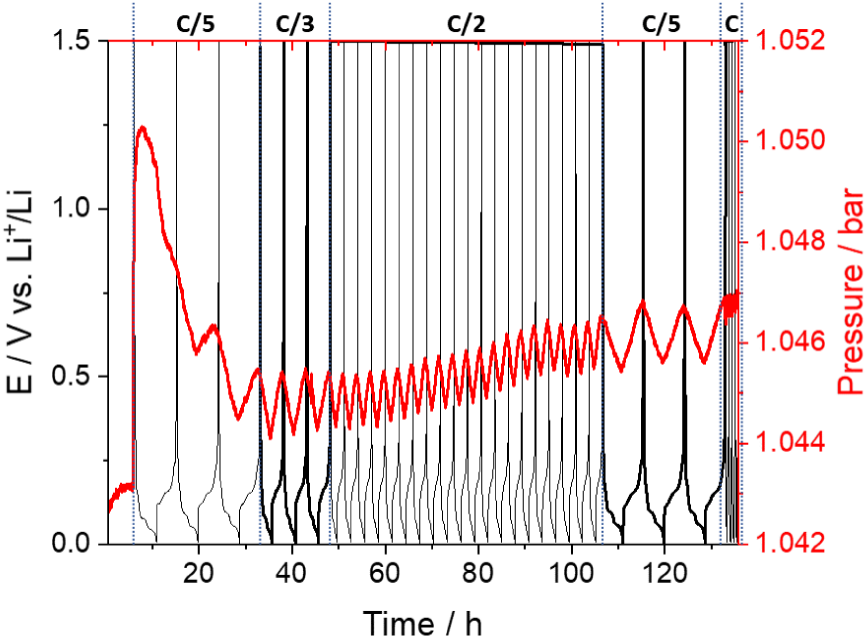
As in Figure 1, but with a graphite vs lithium cell in which the lithium electrode was not presoaked in the electrolyte and with a rest period for cell equilibration of only 6 h.

The operando pressure measurements presented in Figure 1 were obtained using a lithium counter-electrode that had been presoaked in the electrolyte for 24 h, and additionally, the cell was equilibrated for 48 h with the graphite electrode held at a potential of 1.5 V vs Li^+^/Li. This additional soaking step and rest period were introduced to enable the full reaction of the lithium counter electrode with the electrolyte and thus promote its passivation. However, when the measurements were done with untreated lithium electrodes and with a shorter equilibration time of 6 h, the evolution of the cell pressure with cycling was substantially different, as shown in Figure 2.

The operando pressure measurements in Figure 2, of a graphite vs lithium cell with a nonpretreated lithium electrode, show the drastic increase in pressure in the first charging of the graphite, due to gases evolved in the SEI formation, as well as the cyclic and reversible changes in pressure associated with the electrodes’ volume changes during subsequent cycling. These two features were also observed in the operando pressure measurements in Figure 1, done with a graphite vs lithium cell with a pretreated lithium electrode. However, in Figure 2, a marked decrease in pressure is observed after formation (i.e., after the first charge cycle), which is due to the consumption of the gases that were formed in the SEI formation process. Figure S4 shows that these measurements are reproducible, although the magnitude of the pressure buildup shows significant cell-to-cell variability, which we ascribe to potential contamination effects from using a lithium half-cell configuration to study the graphite SEI. However, the rate of gas consumption is found to be reproducible and close to ∼0.04 h^–1^ (see Figure S5). In our previous work,^41^ we employed a longer cell equilibration time of 12 h after cell assembly, and the operando pressure measurements of graphite vs lithium cells showed a small, yet visible, contribution from gas consumption, which we overlooked at that time, but that reflects a slower gas consumption rate at the more passivated lithium counter electrode.

**Figure 3:**
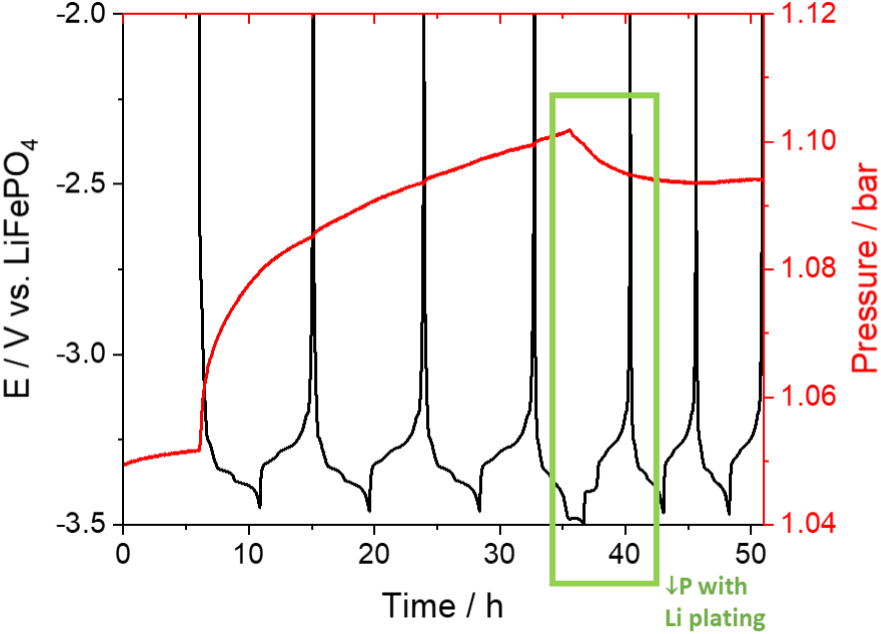
As in Figure 1, but with a graphite vs oversized LiFePO_4_ cell, cycling at a C-rate of C/5 between potentials corresponding to 1.45 V and 5 mV vs Li^+^/Li, except for the 4th cycle, in which a lower potential limit of −50 mV vs Li^+^/Li was used.

In order to investigate the cause of the unexpected decrease in pressure after formation, obtained in graphite vs lithium cells with a nonpretreated lithium electrode, additional operando pressure measurements were performed using an oversized LiFePO_4_ as the counter electrode, as shown in Figure 3. Since the potential of LiFePO_4_ is 3.45 V vs Li^+^/Li when partially delithiated,^48^ a lower potential limit of −3.445 V vs LiFePO_4_ was used in these experiments for the first three cycles, which corresponds to a potential of 0.005 V vs Li^+^/Li, as used in Figures 1 and 2.

The operando pressure measurements in Figure 3, obtained in a graphite vs oversized LiFePO_4_ cell, show a marked increase in pressure in the first charge of the graphite due to the gases produced in the SEI formation process, as in Figure 1 for a graphite vs pretreated lithium cell. Note that these measurements were performed with a cell that had a smaller headspace volume, and consequently, the observed changes in pressure were bigger, as expected from eq 2. The slower rate of buildup of pressure, compared to the results in Figure 1, can be tentatively ascribed to a higher reaction inhomogeneity induced by kinetic limitations at the oversized LiFePO_4_ counter electrode, which was prepared in-house. On the other hand, in contrast with the results in Figure 1, the cyclic reversible changes in pressure due to changes in electrodes’ volume are not clearly visible in Figure 3, because in this case, the changes in electrodes’ volume are smaller and compensate each other (1.2 and −0.9 μL for graphite and LiFePO_4_ electrodes, respectively, resulting in an estimated pressure change of only 0.2 mbar; see details of calculations in the Supporting Information), and therefore the small, associated change in pressure is buried in the large pressure increase due to gases evolved in the process of SEI formation.

The absence of a marked decrease in pressure after formation for the graphite vs LiFePO_4_ cell in Figure 3, which is seen for the graphite vs nonpretreated lithium cells in Figure 2, suggests that such a decrease in pressure is due to the reactivity of the lithium electrode in the consumption of SEI-formed gases. This was then confirmed by performing a charge cycle (fourth cycle in the same graphite vs LiFePO_4_ in Figure 3, highlighted with a green box) in which the graphite was polarized to a low potential of −3.5 V vs LiFePO_4_ (equivalent to −0.05 V vs Li^+^/Li) to induce lithium plating on the graphite electrode. A clear decrease in pressure could be observed that was triggered by the process of lithium plating on graphite, thus confirming that nonpretreated lithium metal consumes the gases that are produced as products of the SEI formation on graphite. Note that a decrease in the rate of gas evolution would not produce a pressure decrease since the operando pressure measurements are done in sealed cells, and thus, the gases accumulate inside the cell. Although a few studies have reported the evolution of gases as a result of lithium plating (due to decomposition reactions of the electrolyte in contact with the newly formed lithium surfaces),^15,49,50^ the present observation of the decrease in pressure due to lithium plating is unexpected.

To shed light into the nature of the gas consumption reaction, the composition of the gas produced during cell cycling was determined by connecting the cell to a mass spectrometer via an online electrochemical mass spectrometry setup (OEMS).^42^ A very thin capillary was used to limit the flow of gases, from the cell to the mass spectrometer, to a low value of 9 μL min^–1^ (see details of the determination of the flow rate and associated equations in Figure S6), thus minimizing perturbance of the cell reactions by the measurements. A pressure controller, connected to an argon supply, was used to keep the internal pressure of the cell constant (Figure S7).

**Figure 4:**
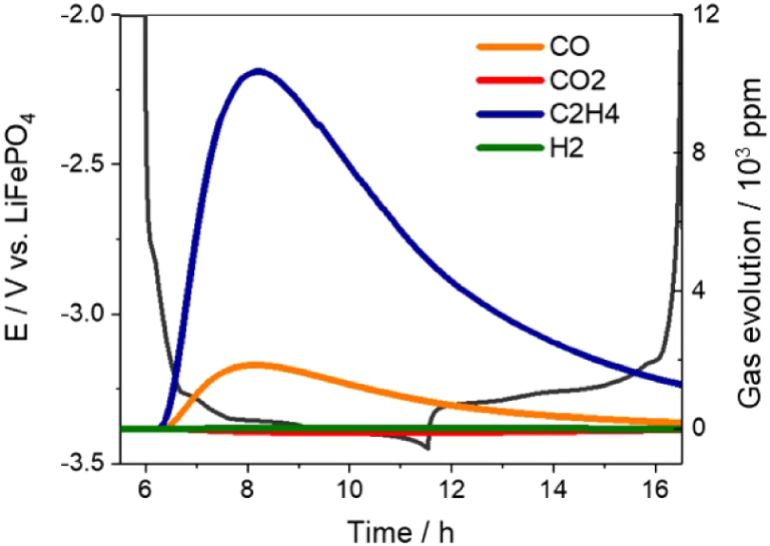
Results of the analysis of gases evolved from a graphite vs LiFePO_4_ cell using the OEMS system shown in Figure S7 and the experimental conditions in Figure 3.

The results of the analysis of gases from a graphite vs LiFePO_4_ cell using the OEMS setup are shown in Figure 4, and Figure S8 shows that the same gases are also formed in a graphite vs lithium cell. The main gases formed are C_2_H_4_ and CO, in agreement with previous gas analysis studies on the graphite SEI formation.^12,43^ Our results show that the C_2_H_4_ and CO signals peak in intensity and then slowly decrease over time. The rate of decrease of the signals (of around ∼0.25 h^–1^) is in agreement with the expected rate of removal of gases from the cell through the capillary (with a flow of the Ar carrier gas of ∼9 μL min^–1^ over a cell headspace volume of ∼3 mL, giving an estimated removal rate of around ∼0.18 h^–1^). Due to the removal of the gases from the cell, the study of the gas consumption reaction (which is slower, with a reaction rate of around ∼0.04 h^–1^, Figure S5) is difficult, and thus, the operando pressure measurements (which are done in a closed cell) are better suited for that purpose.

The results in Figures 4 and S8 also show that the signals due to other gases (H_2_ and CO_2_) are very small/negligible, which confirms that the amount of water contamination in our system is minimal. The reduction of water on graphite electrodes produces H_2_ and hydroxide ions,^12^ and in addition, the presence of water and hydroxide ions promotes the decomposition of the electrolyte forming CO_2_.^45,51^ None of these undesirable side-reactions occur to a significant extent under our experimental conditions.

The OEMS gas analysis in Figures 4 and S8 demonstrates that C_2_H_4_ is, by far, the main gas evolved in the first charge cycle of graphite electrodes. Integration of the C_2_H_4_ signal during the duration of the measurements gives a total volume of C_2_H_4_ evolved, normalized by the mass of graphite, of ∼1.7–1.8 mL/g (see details of calculations in Supporting Information), in reasonable agreement with the value of ∼1.6 mL/g obtained from the operando pressure measurements in Figure 1. Although CO is also evolved, the signal is around a factor of 5 less intense. On the other hand, in the operando pressure measurements done in graphite cells with nonpretreated lithium electrodes (Figures 2 and S4), the decrease in the cell pressure after formation, due to the consumption of SEI-formation gases by the lithium electrode, was very marked, reaching a decrease of more than 50% of the gases produced initially in the SEI formation process. Consequently, such dramatic consumption of gases cannot be due to the consumption of CO only, and thus the present results compellingly demonstrate that C_2_H_4_ must be consumed in nonfully passivated lithium electrodes.

Previous work by Dahn’s group reported a slow decrease in the volume of Li-ion pouch cells due to gas consumption.^52^ Their experiments were done in NMC/graphite cells, and the analysis of the gases by gas chromatography showed that C_2_H_4_ was the main gas product, from which they concluded that C_2_H_4_ was slowly consumed at the graphite electrode, and the formation of polyolefins was tentatively suggested as the C_2_H_4_ consumption reaction product. Further work by Dahn’s group confirmed, via XPS measurements, that the graphite electrodes in NMC/graphite cells that had not degassed exhibited a higher content of carbonaceous compounds (e.g., polyolefins) than those from degassed cells.^53^ Interestingly, the results here presented show that the reactivity of lithium metal anodes toward C_2_H_4_ consumption is much higher than that of graphite, since, without additives, hardly any gas consumption was detected in graphite cells at 25 °C.^52^Figure S9 shows a possible reaction mechanism for the C_2_H_4_ consumption reaction at negative electrodes, forming polyethylene via radical polymerization. A recent investigation^54^ of the surface composition of lithium electrodes that had been in contact with ethylene gas demonstrated the formation of electrochemically inactive species LiH and Li_2_C_2_, which is also in agreement with the present results. The present results also show that using nonpretreated lithium counter-electrodes for gas analysis studies is unsuitable, unless they are gastight sealed in a separated cell compartment,^12,43^ since some gases might not be (fully) detected due to their consumption by the lithium electrode.

The evaluation of the consequences of C_2_H_4_ reactivity on battery anodes in terms of battery performance and safety certainly deserves further studies. To the best of our knowledge, this is the first article demonstrating the direct consumption of C_2_H_4_ upon reaction with lithium electrodes as well as the quantification of the reaction rate. However, the polyolefin/LiH/Li_2_C_2_ coating that could be formed from such a reaction would significantly alter the lithium anode interfacial properties. For example, previous work has shown that coating lithium metal electrodes with polyolefins formed via the polymerization of tetramethylethylene produced significant performance improvements.^55^ Furthermore, since C_2_H_4_ is evolved as a result of SEI (re)formation, understanding its reactivity with battery anodes will also be very helpful for guiding the design of optimal protocols for degassing batteries after the formation cycle as well as the design of mitigation strategies to prevent swelling of faulty or abused batteries. A recent gas analysis of a commercial Li-ion cell demonstrated that C_2_H_4_ evolution is vastly accelerated at high currents,^40^ which are the conditions in which lithium plating is more likely to occur, and thus the reactivity of C_2_H_4_ with metallic lithium is directly relevant to improving commercial Li-ion cell performance and safety.

## 4 Conclusions

By combining two gas analysis techniques (operando pressure measurements and online electrochemical mass spectrometry) on cells containing graphite electrodes with three different types of counter-electrode materials (inert LiFePO_4_ electrodes and fully passivated and nonfully passivated lithium metal electrodes), we have shown that the mechanistic understanding of gas evolution from batteries also needs to consider gas consumption processes. Specifically, we have shown that the main gas evolved in the formation of the graphite SEI, ethylene (C_2_H_4_), is rapidly consumed at lithium metal electrodes that are not fully passivated. The results highlight the differences in the reactivity of graphite and lithium metal electrodes, which in turn implies that the composition of the SEI of these two very important anode materials can be significantly different.

While the formation of C_2_H_4_ is usually taken as a signature of SEI formation, or reformation of the SEI after rupture/disruption, this work shows that C_2_H_4_ can also be rapidly consumed in further SEI forming reactions, thus constituting another reaction pathway of SEI formation, with no gas formation, that had been previously overlooked. Importantly, the composition of the SEI formed via this alternative reaction pathway may contain a higher content of polyolefins, and thus the protective and mechanical properties of the SEI formed with C_2_H_4_ reduction could also be significantly different to those without C_2_H_4_ reduction. Understanding these differences could help to design the best strategies for battery degassing after formation, as well as mitigation strategies for swelling of faulty or abused batteries, and thus certainly warrants further investigation.

## Data Availability

The data for this article are available from the University of Southampton at https://doi.org/10.5258/SOTON/D3165.

## Supporting Information Available

Sketches of the experimental setups, additional results, and details of calculations (PDF)
