# Supplementary material for: Differences in Interfacial Reactivity of Graphite and Lithium Metal Battery Electrodes Investigated Via Operando Gas Analysis
Source: J Phys Chem C Nanomater Interfaces. 2024 Aug 6;128(32):13395–401. doi: 10.1021/acs.jpcc.4c03656 (PMC11331504; doi:10.1021/acs.jpcc.4c03656)
Supplement: Supplementary file 1 — jp4c03656_si_001.pdf [file jp4c03656_si_001.pdf]

Supporting information

Differences in Interfacial Reactivity of Graphite and Lithium Metal Battery Electrodes Investigated via Operando Gas Analysis

J. Padmanabhan Vivek[a,b,]*, Nuria Garcia-Araez[a,b,]*

a: University of Southampton, Chemistry, SO17 1BJ, United Kingdom

b: The Faraday Institution, Harwell Campus, Didcot, OX11 0RA, United Kingdom

* vjp1v16@soton.ac.uk (J. Padmanabhan Vivek), n.garcia-araez@soton.ac.uk (Nuria Garcia-Araez)

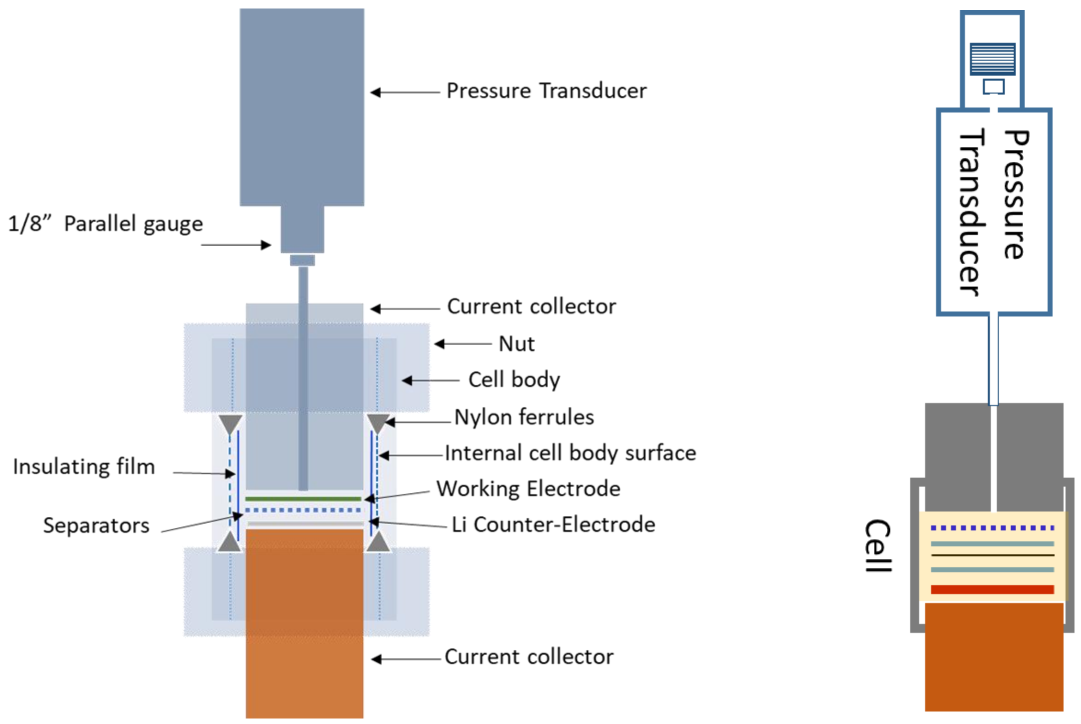

Figure S1. Sketch of the operando pressure cell used in this work. The left graph shows details of all the cell components and the right graph shows a simplified sketch to highlight that the Swagelok cell is directly connected to a pressure transducer (without additional components such as valves, etc.) to achieve a minimal cell headspace volume.

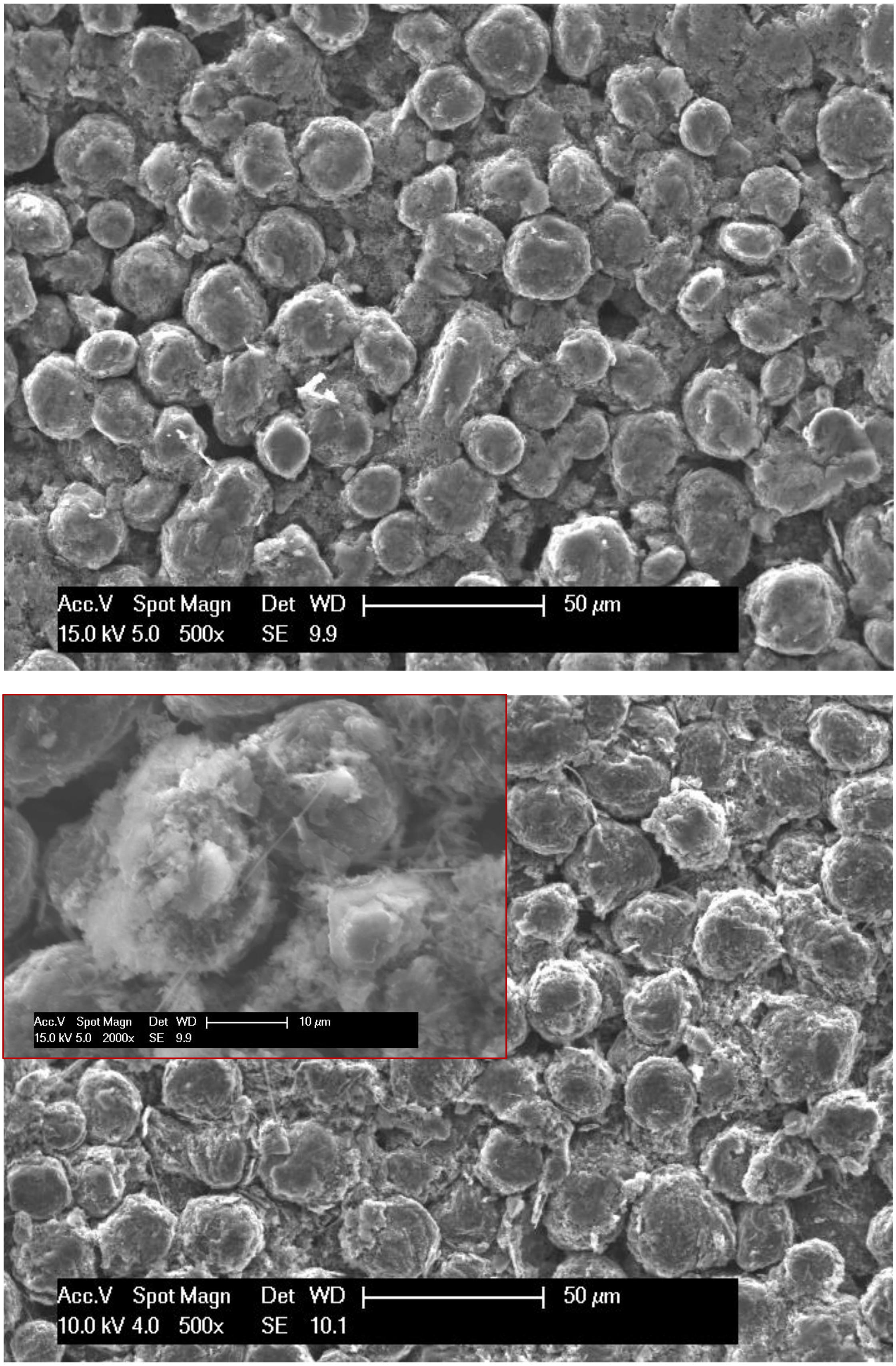

Figure S2. SEM images of mesophase MGP-A graphite electrodes, before (upper graph) and after (lower graph) electrochemical cycling. Images were recorded using a JEOL manufactured JSM59 SEM, with 15 kV accelerating voltage.

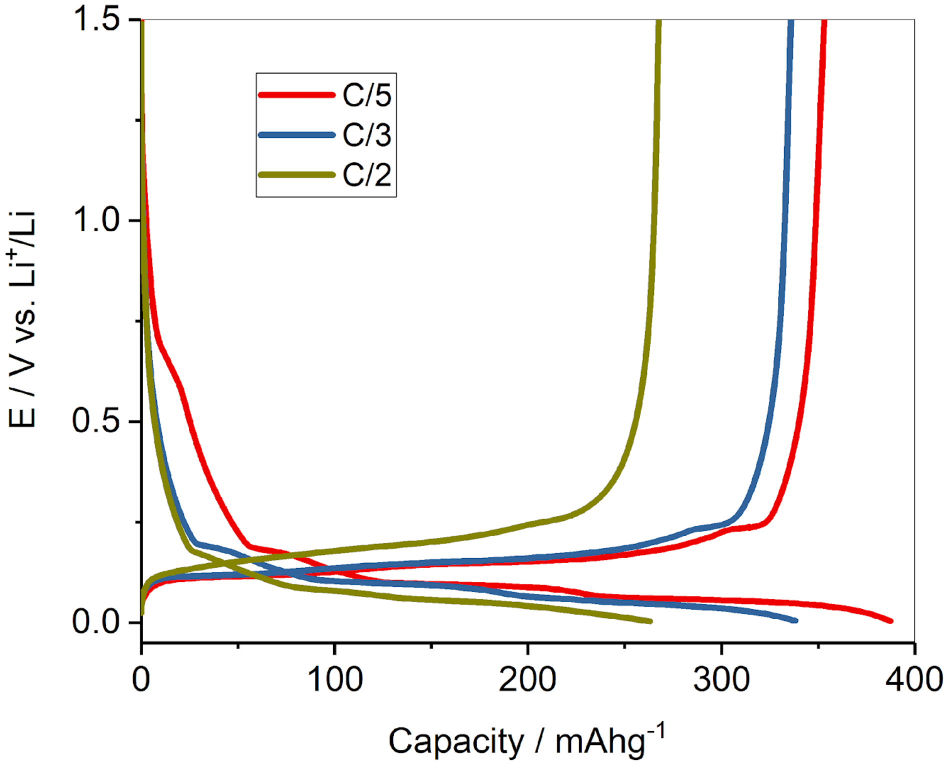

Figure S3. Cycling profile of the mesophase MGP-A graphite electrode in a lithium half-cell.

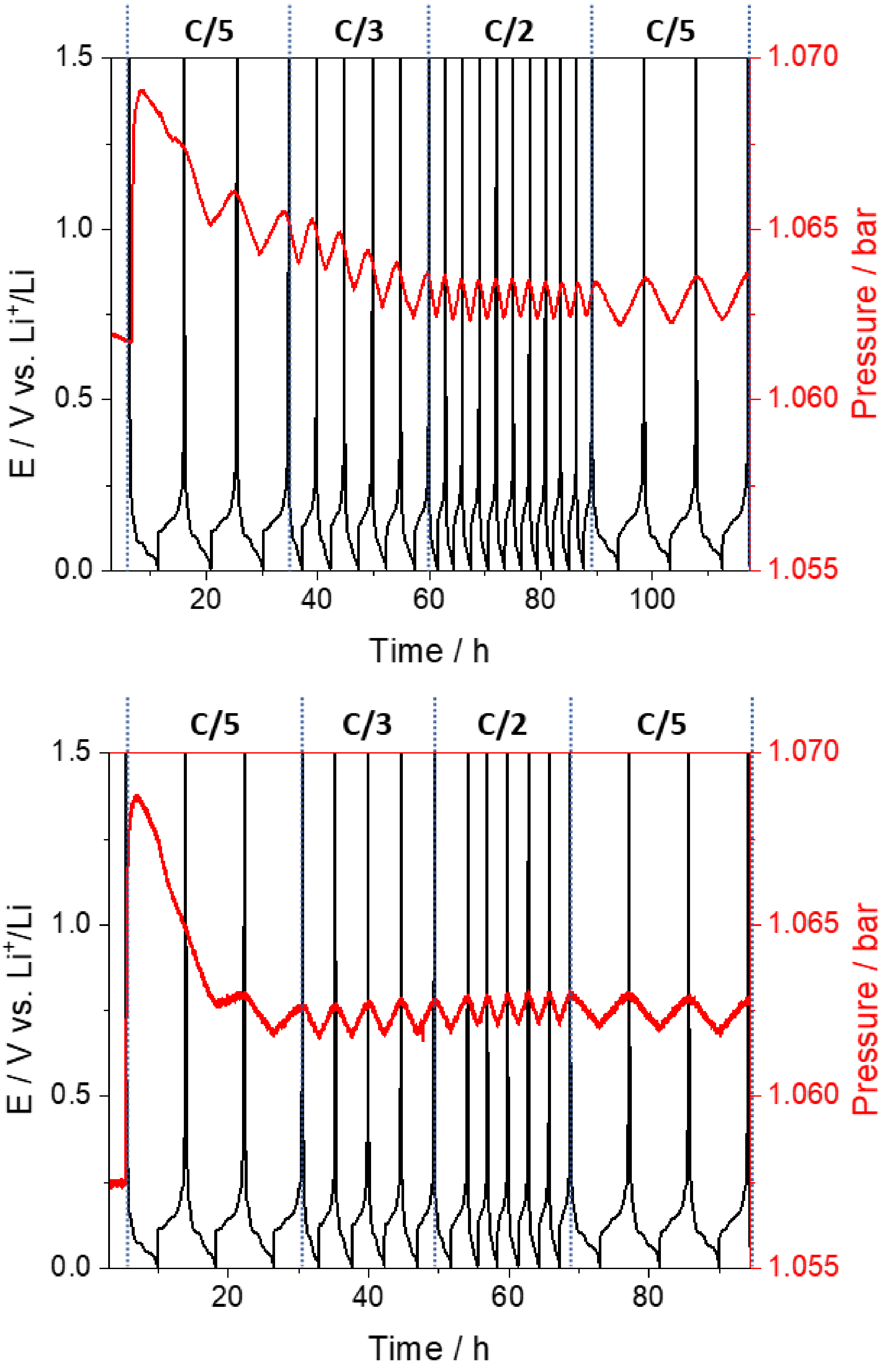

Figure S4. Repeats of the experiment shown in figure 2 in the main article.

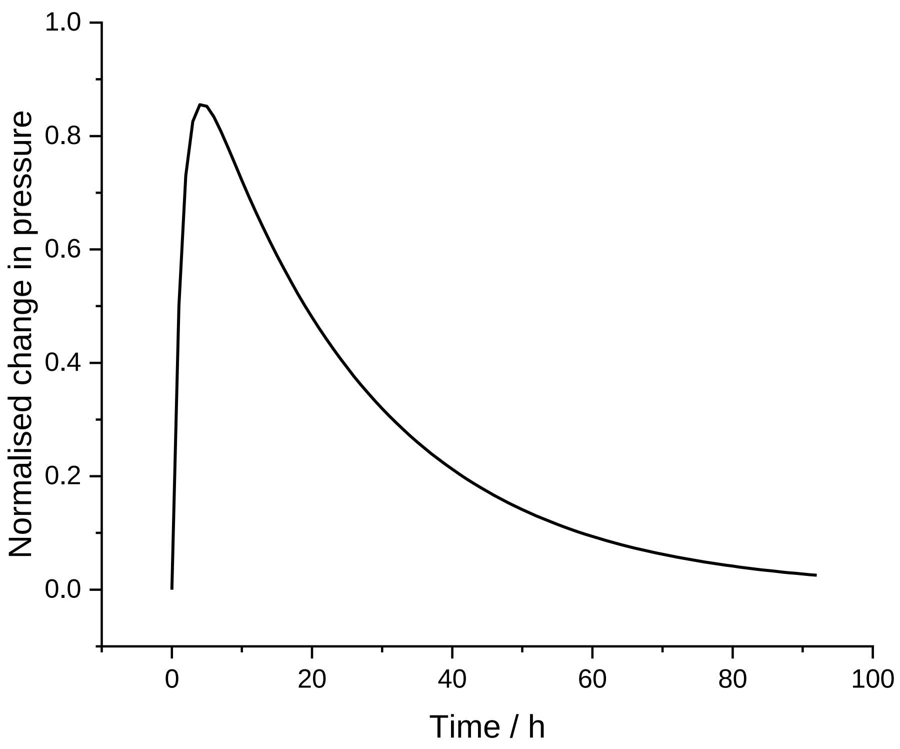

Figure S5. Simulation of the time evolution of the pressure of a system involving two reactions (A →B and B→C), in which only the intermediate product, B, is a gas, and in which the rate constant of the first reaction (A →B) is 0.5 h^{-1} and the rate constant of the second reaction (B→C) is 0.04 h^{-1}. The simulations resemble the time evolution behaviour of the pressure in figures 2 and S4, in which the intermediate product, B, is the C_2H_4 formed in the graphite SEI formation reaction, and then consumed upon reaction with non-pretreated lithium. The value of the rate constant of the first reaction (A →B) is estimated from the time evolution behaviour of the pressure in figure 1, which does not involve the second reaction (B→C) of gas consumption.

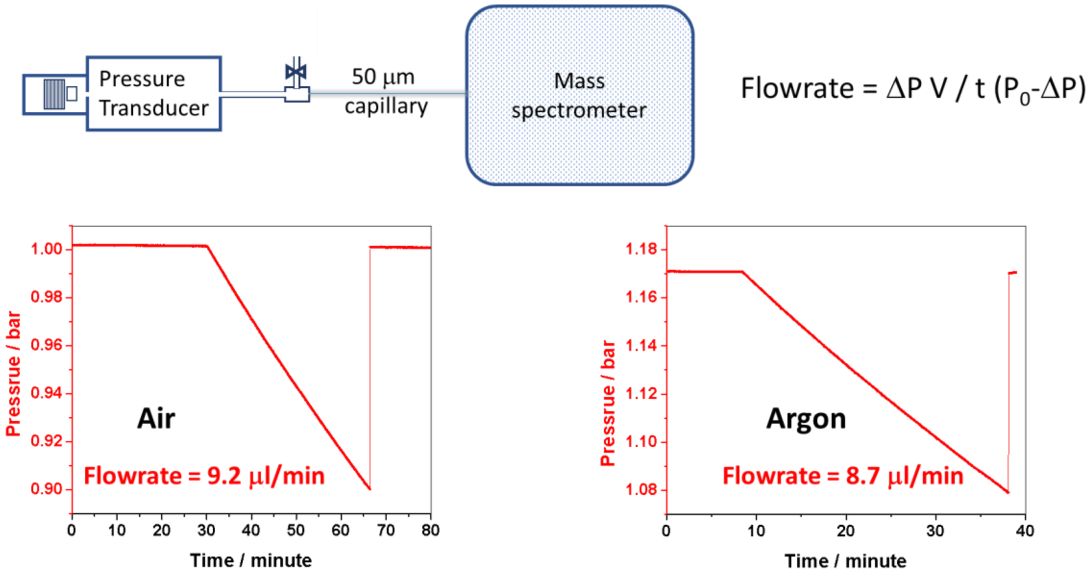

Figure S6. Upper graph: sketch of the set-up employed to estimate the flowrate through a capillary connecting a closed cell to a mass spectrometer. The flowrate is calculated via the equation: flowrate = $\Delta P\ V / [\ t\ (P_0 - \Delta P)]$, where ΔP is the change in pressure inside the closed cell, V is the headspace volume of the closed cell (including the headspace volume of the pressure transducer included to measure the pressure), P_0 is the initial pressure inside the closed cell, and t is time. The lower graphs show the results of the evolution of pressure as a function of time when the cell was filled with air or argon.

Derivation of equation to evaluate the flowrate through a thin capillary connected to a closed cell

For a closed vessel with a headspace volume V and connected to a capillary, the change in pressure inside the vessel, ΔP, is given by:

$$\Delta P\ V = x\ RT$$

where x is the number of moles of gas removed from the cell through the capillary. The flow of gas through the capillary is proportional to the pressure inside the cell:

$$x\ RT = k\ t\ (P_0 - \Delta P)$$

where P_0 is the initial pressure inside the vessel, t is time and k is the flowrate (volume per unit time). Combining the above equations gives:

$$\Delta P\ V\ = k\ t\ (P_0 - \Delta P)$$

And after rearrangement, the following expression for the flowrate is obtained:

$$\text{flowrate} = k = \Delta P\ V / [\ t\ (P_0 - \Delta P)]$$

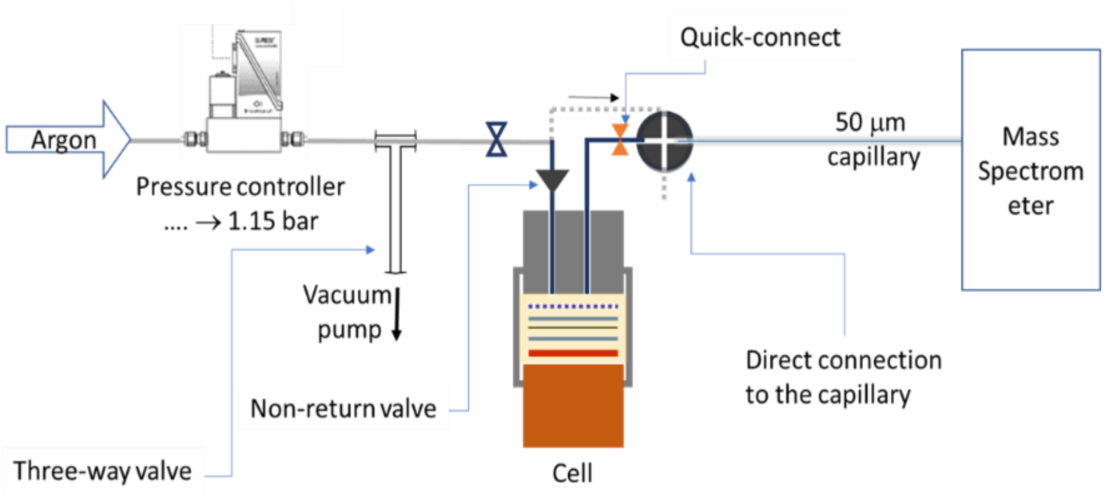

Figure S7. Sketch of the online electrochemical mass spectrometry (OEMS) set-up employed for the analysis of gases evolved from an electrochemical cell containing battery materials. The set-up includes a connection of the cell to a mass spectrometer via a thin capillary that limits the flowrate of the argon carrier gas to ca. 9 µL min^{-1}. A four-port, two-way valve is also placed between the cell and the mass spectrometer to enable a constant flow of argon to the mass spectrometer when the cell is not connected. The cell is also connected to an argon supply via a pressure controller that ensures that the internal pressure of the cell stays constant. A non-return valve is placed between the cell and the pressure controller to avoid gases generated in the cell to diffuse away from the cell to the connections to the pressure controller. In addition, a three-way valve is used to connect the system to a vacuum pump to remove water and other potential contaminants from all connections, prior to the experiment, via application of vacuum.

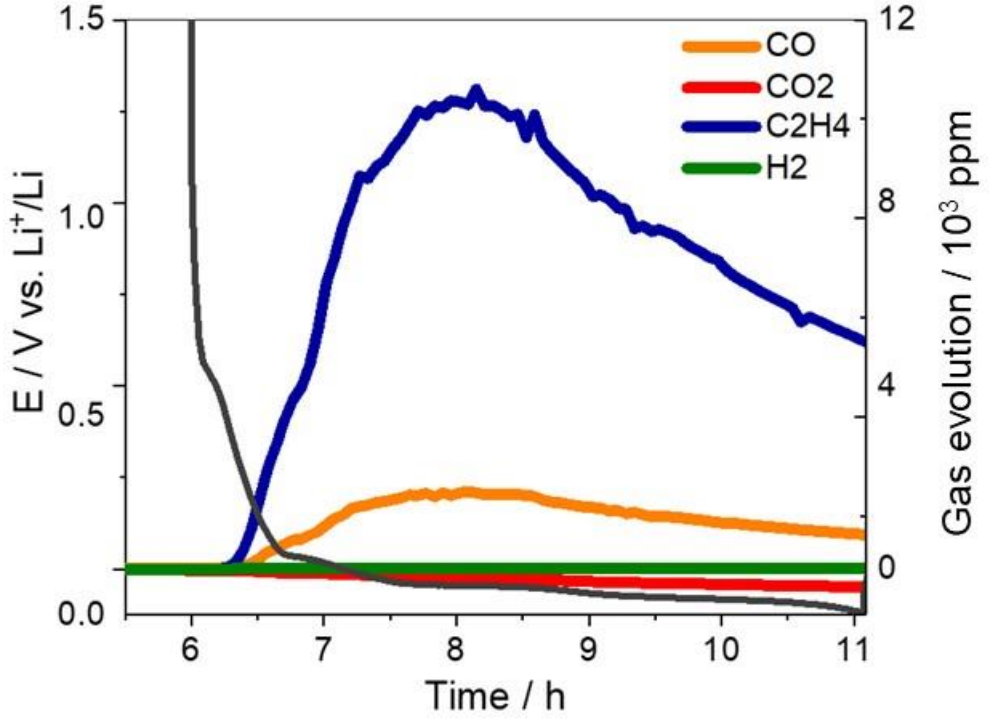

Figure S8. Results of the analysis of the gases evolved from a graphite vs. lithium cell using the OEMS system shown in figure S7, using the same experimental conditions as in figure 2 in the main article.

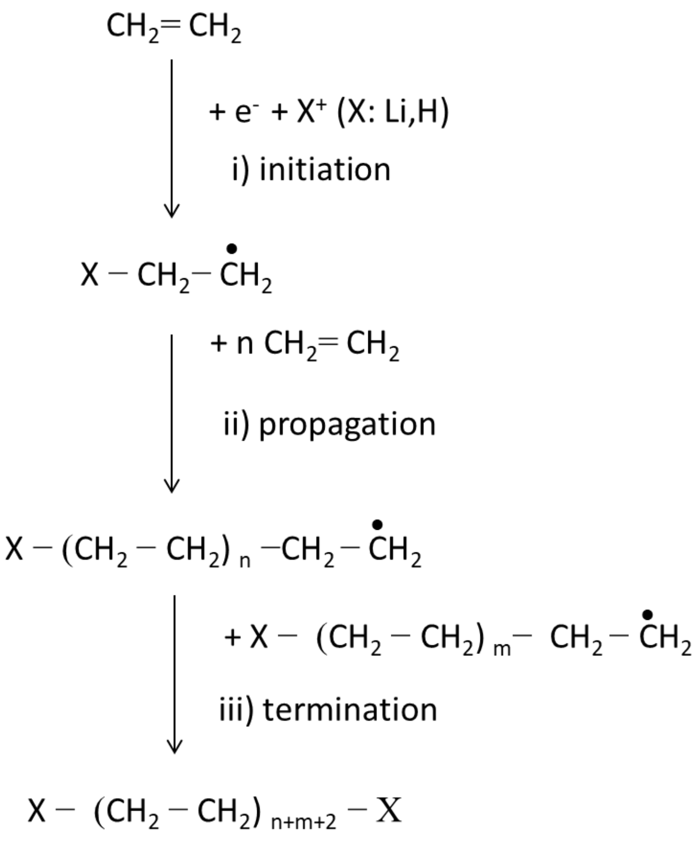

Figure S9. Sketch of a possible reaction pathway for the reduction of C_2H_4 to polyethylene on lithium metal or graphite electrodes.

Calculation of the estimated change in electrodes' volume and internal pressure of the cell due to lithium insertion and extraction reactions.

a) Graphite vs passivated lithium cell shown in figure 1

The mass of graphite in the cell shown in figure 1 is 23.6 mg, and cycling of the cell produces a reversible capacity of 354 mAh g^{-1} at C/5. A volumetric expansion of 13.2% has been detected by XRD for the full lithiation of graphite.[1] Since the theoretical specific capacity of graphite is 372 mAh/g, a volumetric expansion of 354/372*13.2%=12.6% is estimated for the present experimental conditions. Since the graphite density is 2.2 g mL^{-1}, the expected change in volume of graphite is estimated as 0.0236 g / 2.2 g mL^{-1} * 12.6/100 = 0.00135 mL.

The change in volume of the lithium counter-electrode is estimated taking into account that the capacity involved in the process of lithiation of graphite is 9.14 mAh. The mass of lithium required to deliver that capacity is obtained using the theoretical specific capacity of lithium, which equals 3862 mAh g^{-1}, obtaining 9.14 mAh / 3862 mAh g^{-1} = $2.37x10^{-3}$ g. Since the lithium density is 0.534 g mL^{-1}, the volume change of the lithium counter-electrode is estimated as $2.37x10^{-3}$ g / 0.534 g mL^{-1} = 0.00443 mL.

Note that, while the graphite electrode undergoes an expansion, and thus the change in electrode volume is positive, the lithium electrode undergoes a contraction, since lithium is stripped from the lithium electrode, and thus the change in electrode volume is negative. The combined effect of both electrodes produces a total change in volume of 0.00135 mL – 0.00443 mL = –0.00309 mL. Then, the associated change in pressure, ΔP, can be obtained from (equation (2) in the main text):

$$\Delta P = P_0\, \Delta V / (V_{cell} - \Delta V) \quad \text{(S1)}$$

where P_0 is the initial pressure (in this case, around 1.063 bar) and V_{cell} is the cell headspace volume (in this case, 2.55 mL), giving an estimated value of ΔP= -1.3 mbar for the process of lithium insertion into graphite in a graphite vs. lithium cell.

b) Graphite vs $LiFePO_4$ cell shown in figure 3

Following the same method of calculation described above, and taking into account that the mass of graphite in the cell is 20.8 mg and that the experimental specific capacity is 355 mAh g^{-1}, the change in volume of graphite is estimated as 0.00119 mL. Then, the change in volume of the counter-electrode is estimated taking into account that the capacity involved in the process of lithiation of graphite is 8.06 mAh. Using the theoretical capacity of $LiFePO_4$ of 170 mAh g^{-1}, the mass of $LiFePO_4$ that needs to be oxidized to $FePO_4$ to provide the required capacity of graphite lithiation is estimated as 8.06 mAh/ 170 mAh g^{-1} = 0.047 g. XRD measurements have shown that the transformation of $LiFePO_4$ into $FePO_4$ produces a change of 6.5% in unit cell volume when referred to the initial state $LiFePO_4$, and that the $LiFePO_4$ density is 3.6 g mL^{-1}.[2] Therefore, the expected change in $LiFePO_4$ electrode volume for the present conditions is estimated as 0.047 g / 3.6 g mL^{-1} * 6.5/100 = 0.00086 mL.

Note that, while the graphite electrode undergoes an expansion, and thus the change in electrode volume is positive, the $LiFePO_4$ electrode undergoes a contraction, and thus the change in electrode volume is negative. The combined effect of both electrodes produces a total change in volume of 0.00119 ml – 0.00086 ml = 0.00033 ml. Then, the associated change in pressure, ΔP, can be obtained from equation (S1), taking into account that the cell headspace volume is ca. 1.6 mL in this case, thus giving a value of ΔP= 0.2 mbar.

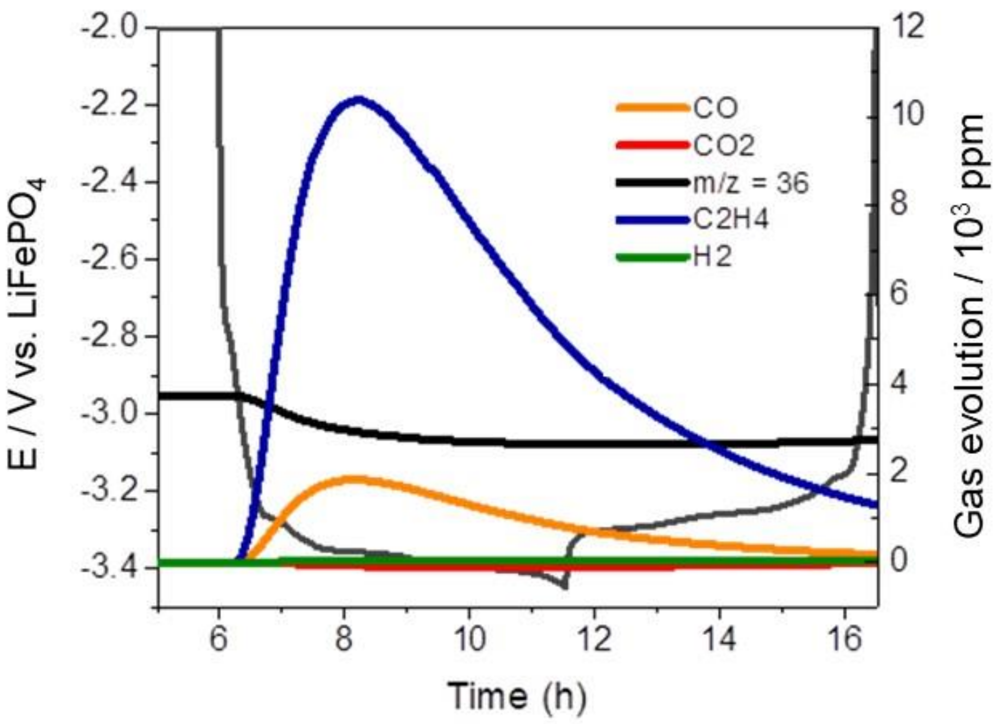

Figure S10. As in figure 4 in the main text but including the results of the signal at m/z = 36, which corresponds to the ^{36}Ar isotope that has a natural abundance of 0.34% and thus it is contained in the Ar carrier gas (whose main isotope is ^{40}Ar that has a natural abundance of 99.6%).[3] The ^{36}Ar isotope signal is measured because it has a concentration similar to the gases formed during battery cycling (e.g. C_2H_4), while it allows monitoring variations in the partial pressure of the Ar carrier gas inside the cell. The small decrease of the ^{36}Ar isotope signal at the beginning of cycling is due to the formation of other gases inside the battery, and the consequent decrease in the argon partial pressure, produced by the pressure controller (see experimental set-up in figure S7), so as to maintain the pressure inside the cell constant.

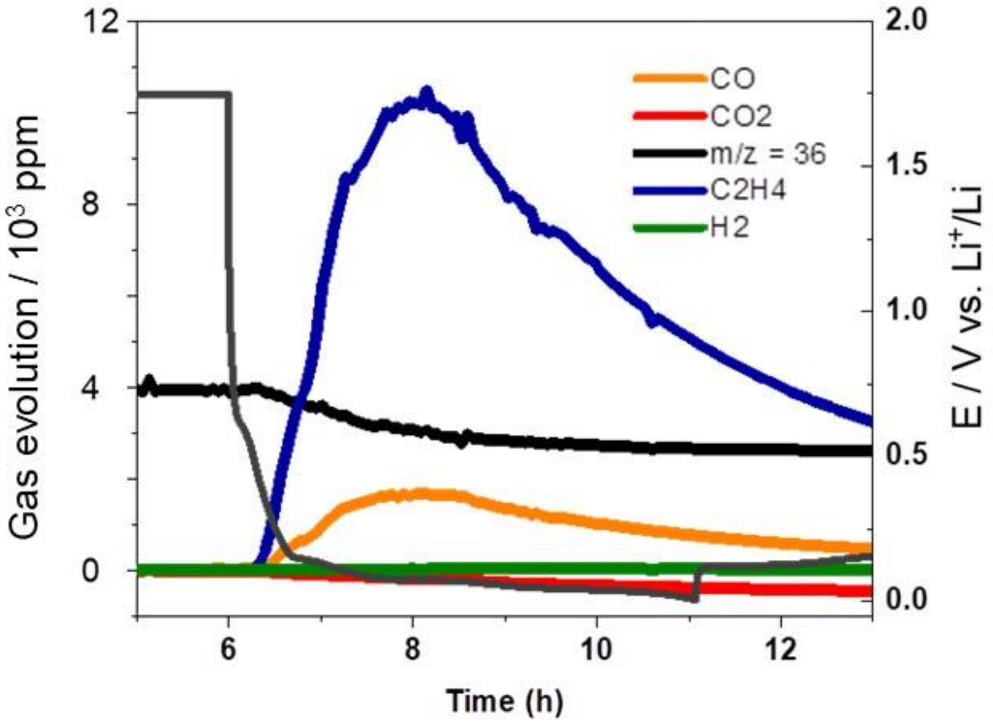

Figure S11. As in figure S8 but including the results of the signal at m/z = 36, which corresponds to the ^{36}Ar isotope that has a natural abundance of 0.34%.[3] As discussed in the caption of figure S10, the ^{36}Ar isotope signal shows a small decrease at the beginning of cycling due to the formation of other gases inside the cell, for which the pressure controller (see experimental set-up in figure S7), decreases the Ar partial pressure so as to maintain the cell pressure constant.

Calculation of the total volume of ethylene gas evolved from graphite electrodes using OEMS measurements

The formation ethylene gas (C_2H_4) is monitored with the mass spectrometry signal at m/z=26, which, after calibration, provides the concentration of C_2H_4 in ppm. The integration of the C_2H_4 gas evolution signal, in ppm, during the duration of the OEMS measurements in figure 4 gives a value of ~52,000 ppm h, which divided by the duration of the measurements, 10 h, gives a time-averaged concentration of ~5,200 ppm. The total volume of gas that contained the C_2H_4 gas in these measurements equals the cell headspace volume, ~3 mL, plus the additional volume of Ar supplied to the cell to maintain the cell pressure constant, and the latter is obtained as the product of the duration of the experiment, 10 h, and the Ar flow rate, ~9 µL min^{-1}, giving a total gas volume of ~3 mL + 5.4 mL = ~8.4 mL. The total C_2H_4 gas volume is, then, obtained as ~5200 x 10^{-6} * 8.4 mL = ~0.044 mL, which normalised by the mass of graphite gives ~1.8 mL/g. Using

the same method of calculation with the results in figure S8, a total volume of C_2H_4 normalised by the mass of graphite of ~1.7 mL/g is obtained.

References

(1) Schweidler, S.; De Biasi, L.; Schiele, A.; Hartmann, P.; Brezesinski, T.; Janek, J. Volume Changes of Graphite Anodes Revisited: A Combined Operando X-Ray Diffraction and in Situ Pressure Analysis Study. *J. Phys. Chem. C* **2018**, *122* (16), 8829–8835.

(2) Padhi, A. K.; Nanjundaswamy, K. S.; Masquelier, C.; Okada, S.; Goodenough, J. B. Effect of Structure on the Fe3 + / Fe2 + Redox Couple in Iron Phosphates. *J. Electrochem. Soc.* **1997**, *144* (5), 1609–1613.

(3) David R. Lide, ed., CRC Handbook of Chemistry and Physics, Internet Version 2005, <http://www.hbcpnetbase.com>, CRC Press, Boca Raton, FL, 2005.
